# Effect of mistletoe extract on tumor response in neoadjuvant chemoradiotherapy for rectal cancer: a cohort study

**DOI:** 10.1186/s12957-021-02293-4

**Published:** 2021-06-15

**Authors:** Jeong-Heum Baek, Youngbae Jeon, Kyoung-Won Han, Dong Hae Jung, Kyung-Ok Kim

**Affiliations:** 1 Division of Colon and Rectal Surgery, Department of Surgery, Gil Medical Center, Gachon University College of Medicine, Namdong-daero 774 beon-gil, Namdong-gu, Incheon, 21565 Republic of Korea; 2 Department of Pathology, Gil Medical Center, Gachon University College of Medicine, Incheon, Republic of Korea; 3 Gachon Medical Research Institute, Gil Medical Center, Gachon University, Incheon, Republic of Korea

**Keywords:** Rectal cancer, Chemoradiation, Tumor response, Abnoba Viscum, *Viscum album*, Mistletoe

## Abstract

**Background:**

Mistletoe extract, used as a complementary chemotherapeutic agent for cancer patients, has anticancer effects against various malignancies. The aim of the present study was to evaluate the effect of mistletoe extract (Abnoba Viscum Q®) on tumor responses to neoadjuvant chemoradiotherapy (NCRT) for locally advanced rectal cancer.

**Methods:**

This study included patients with rectal cancer who underwent NCRT between January 2018 and July 2020. In the mistletoe group (MG), the patients were administered Abnoba Viscum Q® subcutaneously during chemoradiotherapy—maintained just before surgery. Patient demographics, clinical outcomes, histopathological outcomes, and terminal deoxynucleotidyl transferase-mediated dUTP-biotin nick-end labeling (TUNEL) assay results were compared between the MG and non-mistletoe group (NMG). Two rectal cancer cell lines (SNU-503 and SNU-503R80Gy) were treated with Abnoba Viscum Q® to assess its mechanistic effects in vivo.

**Results:**

Overall, the study included 52 patients (MG: n = 15; NMG: n = 37). Baseline demographics between the two groups were similar, except carbohydrate antigen 19-9 levels and tumor location from the anal verge. There was no difference in the clinical stage between the two groups. A better tumor response in the MG, relative to the NMG, was observed with respect to tumor regression grade (TRG), T stage, and overall tumor–node–metastasis stage. Tumor response was significantly better in the MG than in the NMG in terms of pathologic complete response rate (53.3% vs. 21.6%, *P* = 0.044), good TRG response (66.7% vs. 32.4%, *P* = 0.024), T downstaging (86.7% vs. 43.2%, *P* = 0.004), and overall downstaging (86.7% vs. 56.8%, *P* = 0.040). The toxicities during NCRT were minimal in both groups. More apoptotic cells were noted in MG samples than in the NMG samples on TUNEL staining. Cleaved caspase-3 level following treatment with Abnoba Viscum Q® was higher in SNU-503R80Gy cells than in SNU-503 cells.

**Conclusion:**

Patients treated with chemoradiation combined with mistletoe extract showed better outcomes than patients not treated with mistletoe extract in terms of tumor responses. This diversity in treatment may improve the efficacy of NCRT, leading to better oncologic outcomes. Prospective and randomized studies with long-term follow-up are warranted to confirm and extend these results.

## Background

Colorectal cancer, one of the most common solid tumors, is the third leading cause of cancer-related deaths and the second leading cause of mortality amongst malignancies worldwide [1]. The standard treatment for patients with locally advanced rectal cancer is neoadjuvant chemoradiotherapy (NCRT) before radical surgery to reduce locoregional recurrence and the toxicity of chemoradiation [2, 3]. Tumor downstaging and pathologic complete response (pCR) after NCRT for rectal cancer are associated with good survival outcomes when compared with non-response to NCRT [4–8]. The usefulness of neoadjuvant chemotherapy for rectal cancer has been explored to avoid NCRT-associated radiation toxicity. However, its effect on tumor downstaging or pCR has not been better than that of NCRT [9].

Although NCRT is associated with reduced locoregional recurrence of rectal cancer, some patients develop locoregional recurrence, leading to poor oncologic outcomes. Therefore, it is essential to enhance the tumor response to attain good oncological results in such patients. Attempts have been made to introduce more toxic chemotherapeutic agents and biologics to the conventional NCRT regime. Studies have investigated the effects of oxaliplatin or targeted agents added to NCRT in patients with advanced rectal cancer. Unfortunately, these agents did not improve the tumor response and were more toxic than NCRT alone [10–13]. New regimens with less toxicity and good compliance are needed to enhance tumor response and complete remission rate in patients with rectal cancer.

Mistletoe (scientific botanical name: *Viscum album* L.) is a semi-parasitic plant belonging to the order Santalales. Extracts from European mistletoe are widely used as complementary and alternative agents to treat patients with various malignancies, including colorectal, lung, oral, pancreatic, and breast cancers. The active molecules in these extracts, including viscotoxin and lectin, exhibit antitumor effects because of direct cytotoxicity and upregulation of cell-mediated immunity [14, 15]. Mistletoe extracts are safe and help improve the quality of life of cancer patients during chemotherapy [16–18]. Although the antitumor effects and safety have been reported, no clinical studies with mistletoe extract have been undertaken to understand their effect on tumor regression when administered during NCRT for rectal cancer. Therefore, in the present study, we investigated the effect of mistletoe extract on the tumor response to NCRT in patients with locally advanced rectal cancer.

## Methods

### Patient selection

This single-center retrospective observational study included 52 consecutive patients with clinical stage II–III rectal adenocarcinoma treated with long-course NCRT between January 2018 and July 2020 at Gil Medical Center, a tertiary referral hospital in Korea. Mistletoe extract was administered during NCRT, and patients were stratified into two groups: those who received mistletoe extract (mistletoe group; MG) and those who did not (non-mistletoe group; NMG). Patients with incomplete NCRT were excluded from the study. The conditional search of patient information was undertaken by implementing a clinical research data warehouse system, and detailed data were collected from electronic medical records at Gil Medical Center. The Institutional Review Board and Ethics committee of our hospital approved the study (approval no. GFIRB2020-304).

### Neoadjuvant chemoradiotherapy (NCRT)

All 52 patients were treated with long-course NCRT according to the National Comprehensive Cancer Network guidelines. Patients were administered pelvic radiotherapy with 50.4 Gy of radiation in 28 fractions over 5 weeks. The dosing schedules for concurrent chemotherapy were as follows: capecitabine (825 mg/m^2^, orally twice daily) for 5 days per week or 5-fluorouracil (5-FU; 400 mg/m^2^, intravenous bolus) with leucovorin (20 mg/m^2^, intravenous bolus) for 4 days during weeks 1 and 5 of NCRT.

### Mistletoe extract

Abnoba Viscum Q® (ABNOBA GmbH, Pforzheim, Germany) was used as the mistletoe extract. There was no criterion for selecting patients to be treated with Abnoba Viscum Q®. The extract was administered subcutaneously at a dose of 0.02 mg/ampule thrice a week for 3 weeks, followed by 0.2 mg/ampule thrice a week for 3 weeks, 2 mg/ampule thrice a week for 3 weeks, and, finally, 20 mg/ampule thrice a week; thereafter, a final dose of 20 mg was administered just before surgery. The patients self-administered Abnoba Viscum Q® after learning how to inject it from the medical staff and were followed up at our outpatient clinic. The administration of the extract was transiently halted in case of any local or systemic adverse events, such as severe urticaria, local swelling > 5 cm, or high fever > 38 °C.

### Terminal deoxynucleotidyl transferase (TdT)-mediated dUTP-biotin nick-end-labeling (TUNEL) assay

Tissue samples were obtained from patients in the MG and the NMG after curative surgery to assess apoptosis using the TUNEL assay. Slides were prepared from frozen tissue and fixed with a paraformaldehyde solution. Following fixation, the TdT reaction mixture was added to the slides. The slides were then immersed in hydrogen peroxide with a streptavidin–horseradish peroxidase. Finally, 3,3-diaminobenzidine tetrachloride solution was added and the number of TUNEL-stained cells was counted under an optical microscope.

### Western blot analysis

Two rectal cell lines (SNU-503 and SNU-503R80Gy, Seoul National University Korean Cell Line Bank, SNU-503R80Gy: A total of 80Gy of fractionated ionizing radiation to SNU-503 over 40 times by using Cesium-137 irradiator) were treated with 20 μg/mL Abnoba Viscum Q®. Forty-eight hours after treatment, the cells were harvested, and 20 μg protein was separated on a 15% sodium dodecyl sulfate-polyacrylamide gel electrophoresis gel. Expression of caspase-3, cleaved caspase-3, and β-actin was confirmed using antibodies against the respective proteins.

### Assessment parameters and statistical analysis

Patient demographics, clinical outcomes, and histopathological outcomes were compared between patients in the MG and the NMG. Clinical tumor–node–metastasis (TNM) stage and mesorectal fascia (MRF) involvement were assessed using computed tomography (CT) and magnetic resonance imaging (MRI). Abdominoperineal CT and rectal MRI were performed before and after NCRT to evaluate the status of the tumor and lymph node. The tumor distance from the anal verge (AV) was measured using MRI. The tumor regression grade (TRG) was measured on a scale of 0 (complete response: no viable cancer cells) to 3 (poor or no response), according to the American Joint Committee on Cancer (AJCC) classification [19]. Response to treatment was graded as good (AJCC grades 0 and 1) or poor (AJCC grades 2 and 3). T, N, and overall TNM downstaging were defined on the basis of downstaging between baseline imaging results and pathologic stage after curative resection. Continuous variables were analyzed using the Mann–Whitney U test, and categorical variables were analyzed using the chi-square test or Fisher’s exact test. Significant variable differences between the two groups were defined as those with *P*-values < 0.05. All statistical analyses were performed using SPSS 23.0 software for Windows (IBM SPSS Inc., Chicago, IL, USA).

## Results

### Baseline patient characteristics

A total of 52 patients were included in the study: 15 in the MG and 37 in the NMG. All patients in the MG received the complete dose of mistletoe extract according to the schedule. The median age of the patients was 68 years (range 38–86 years), and the study group comprised 35 men and 17 women. Baseline demographics were largely similar between the two groups (Table 1), although there were few exceptions. CA 19-9 level was higher in the NMG than in the MG (*P* = 0.01), and the tumor distance from the AV was longer in the NMG than in the MG (*P* = 0.008). MRF involvement was present in 10 (66.7%) patients in the MG and 19 (51.4%) patients in the NMG (*P* = 0.314), based on the abdominoperineal CT and rectal MRI findings. All patients in the MG were diagnosed with clinical stage III disease, whereas the NMG had three (8.1%) patients with clinical stage II disease and 34 (91.9%) patients with clinical stage III disease. There was no statistically significant difference in the clinical stage between the two groups (*P* = 0.548).

**Table 1:** Baseline patient demographics in the mistletoe and non-mistletoe groups

Variables	MG (*n* = 15)	NMG (*n* = 37)	*P*-value
Age (years)	68 (38–83)	67 (46–86)	0.864^†^
Sex			0.525^¶^
Male	9 (60.0)	26 (70.3)	
Female	6 (40.0)	11 (29.7)	
BMI (kg/m^2^)	23.4 (20.8–28.3)	24.4 (15.1–31.5)	0.473^†^
ASA score			0.254^¶^
1	0	4 (10.8)	
2	15 (100)	30 (81.1)	
Unknown	0	3 (8.1)	
Past history			
Diabetes	5 (33.3)	8 (21.6)	0.483^¶^
Cardiovascular disease	7 (46.7)	18 (48.6)	0.897^¶^
Others	5 (33.3)	13 (35.1)	0.902^¶^
Baseline tumor markers			
CEA (ng/mL)	2.9 (0.5–43.7)	2.9 (0.6–44.9)	0.992^†^
CA 19-9 (U/mL)	4.2 (1.2–184.7)	12.1 (1.2–1222.8)	0.010^†,**^
Tumor distance from the AV (cm)	3.6 (0–10.0)	6.0 (2.0–10.0)	0.008^†,**^
MRF involvement			0.314^¶^
Yes	10 (66.7)	19 (51.4)	
No	5 (33.3)	18 (48.6)	
cT before NCRT			0.412^¶^
cT2	1 (6.7)	0	
cT3	12 (80.0)	32 (86.5)	
cT4	2 (13.3)	5 (13.5)	
cN before NCRT			0.086^¶^
cN0	0	3 (8.1)	
cN1	7 (46.7)	6 (16.2)	
cN2	8 (53.3)	28 (75.7)	
Baseline clinical stage (AJCC 8th edition)			0.548^¶^
Stage II	0	3 (8.1)	
Stage III	15 (100)	34 (91.9)	

Data are presented as median (range) for continuous variables and *n* (%) for categorical variables

^†^Mann–Whitney test; ^¶^chi-square test or Fisher’s exact test; ^**^statistical significance (*P* < 0.05)

*MG*, mistletoe group; *NMG*, non-mistletoe group; *BMI*, body mass index; *ASA*, American Society of Anesthesiologist; *CEA*, carcinoembryonic antigen; *CA*, carbohydrate antigen; *AV*, anal verge; *MRF*, mesorectal fascia; *NCRT*, neoadjuvant chemoradiotherapy; *AJCC*, American Joint Committee on Cancer

### Perioperative outcomes

All patients received a total of 50.4 Gy of pelvic radiation. The median interval to surgery after the completion of NCRT was 9 weeks (range 5.9–14.1). The intervals in the MG and NMG were 8.6 weeks and 8.7 weeks, respectively, with no statistically significant difference (*P* = 0.808). All patients were treated with oral capecitabine, except one patient who was treated with 5-FU/leucovorin. With regard to the surgical type, there were 11 low anterior resections, 34 ultra-low anterior resections, two abdominoperineal resections, three Hartmann’s operations, and two transanal excisions. There were no significant differences in the perioperative clinical outcomes between the two groups (Table 2).

**Table 2:** Short-term clinical outcomes of chemoradiation and surgery in the mistletoe and non-mistletoe groups

Variables	MG (*n* = 15)	NMG (*n* = 37)	*P*-value
Dose of radiation (Gy)	50.4	50.4	n/a
Concurrent chemotherapy			1.000^¶^
Capecitabine	15 (100)	36 (97.3)	
5-Fluorouracil	0	1 (2.7)	
cT after NCRT			0.848^¶^
cT0	1 (6.7)	1 (2.7)	
cT2	6 (40.0)	12 (32.4)	
cT3	7 (46.7)	21 (56.8)	
cT4	1 (6.7)	3 (8.1)	
cN after NCRT			0.666^¶^
cN0	7 (46.7)	14 (37.8)	
cN1	6 (40.0)	14 (37.8)	
cN2	2 (13.3)	9 (24.3)	
Interval to surgery (weeks)	8.6 (6.1–12.9)	8.7 (5.9–14.1)	0.808^†^
Type of surgery			0.506^¶^
Low anterior resection	3 (20.0)	8 (21.6)	
Ultra-low anterior resection	10 (66.7)	24 (64.9)	
Abdominoperineal resection	1 (6.7)	1 (2.7)	
Hartmann’s operation	0	3 (8.1)	
Transanal excision	1 (6.7)	1 (2.7)	
Total operative time (h)	3.0 (0.8–4.1)	3.0 (0.2–5.7)	0.428^†^
Estimated blood loss (mL)	20.0 (3.0–150.0)	30.0 (0–250.0)	0.385^†^
Intraoperative transfusion			n/a
Yes	0	0	
No	15 (100)	37 (100)	
Length of hospital stay (days)	8 (4–37)	8 (5–51)	0.676^†^

Data are presented as median (range) for continuous variables and *n* (%) for categorical variables

^†^Mann–Whitney test; ^¶^chi-square test or Fisher’s exact test

*MG*, mistletoe group; *NMG*, non-mistletoe group; *NCRT*, neoadjuvant chemoradiotherapy

### Toxicity outcomes

Toxicities observed during NCRT are shown in Table 3. According to the Common Terminology Criteria for Adverse Events version 3.0, toxicity was stratified from grade 1 to grade 4. The most common toxicity in both groups was proctitis. Nine patients (60.0%) in the MG and ten patients (27.0%) in the NMG had grade 1 proctitis. Grade 2 neutropenia and grade 3 anemia were observed in the NMG, whereas none of the patients in the MG experienced these adverse events. Pruritus with or without skin rash at the injection site occurred in three patients in the MG (20.0%), whereas this adverse event was not seen in any of the patients in the NMG (*P* = 0.021). All other adverse events, including nausea, vomiting, diarrhea, constipation, oral mucositis, and peripheral neuropathy, were of grade 1, and there were no significant differences in the incidences of these adverse events between the two groups.

**Table 3:** Toxicity outcomes in the mistletoe and non-mistletoe groups

Toxicity	CTCAE								*P*-value^*¶*^
	MG (*n* = 15)				NMG (*n* = 37)				
	Grade 1	Grade 2	Grade 3	Grade 4	Grade 1	Grade 2	Grade 3	Grade 4	
Neutropenia	0	0	0	0	0	1 (2.7)	0	0	> 0.99
Anemia	0	0	0	0	0	0	1 (2.7)	0	> 0.99
Thrombocytopenia	0	0	0	0	0	0	0	0	–
Nausea	4 (26.7)	0	0	0	6 (16.2)	0	0	0	0.448
Vomiting	1 (6.7)	0	0	0	0	0	0	0	0.288
Diarrhea	2 (13.3)	0	0	0	5 (13.5)	0	0	0	> 0.99
Constipation	0	0	0	0	4 (2.8)	0	0	0	0.311
Proctitis	9 (60.0)	0	0	0	10 (27.0)	0	0	0	0.054
Oral mucositis	0	0	0	0	1 (2.7)	0	0	0	> 0.99
Peripheral neuropathy	1 (6.7)	0	0	0	0	0	0	0	0.288
Pruritus	3 (20.0)	0	0	0	0	0	0	0	0.021

Data are presented as *n* (%)

^¶^Chi-square test or Fisher’s exact test

*CTCAE*, Common Terminology Criteria for Adverse Events version 3.0; *MG*, mistletoe group; *NMG*, non-mistletoe group

### Evaluation of tumor responses

Estimations of pCR, downstaging, and TRG were assessed from the histopathologic reports of the patients after curative surgery. Table 4 presents the histopathological outcomes after curative resection. There were no significant differences between the two groups with respect to tumor size, histologic grade, ypN stage, distal resection margin, circumferential resection margin involvement, harvested lymph nodes, or perineural invasion. However, the ypT stage was significantly higher in the NMG than in the MG (*P* = 0.005). Moreover, the lymphovascular invasion was significantly more common in the NMG than in the MG (32.4% vs. 13.3%, *P* = 0.04).

**Table 4:** Histopathological outcomes in the mistletoe and non-mistletoe groups

Variables	MG (*n* = 15)	NMG (*n* = 37)	*P*-value
Tumor size (cm)	2.5 (0–4.5)	3 (0–7.7)	0.293^†^
Histologic grade			0.151^¶^
Well-differentiated	0	1 (2.7)	
Moderately differentiated	6 (40.0)	25 (67.6)	
Poorly differentiated	1 (6.7)	2 (5.4)	
Others	8 (53.3)	9 (24.3)	
ypT after NCRT			0.005^¶,**^
ypT0	8 (53.3)	8 (21.6)	
ypT1	0	1 (2.7)	
ypT2	5 (33.3)	4 (10.8)	
ypT3	2 (13.3)	22 (59.5)	
ypT4	0	2 (5.4)	
ypN after NCRT			0.161^¶^
ypN0	13 (86.7)	21 (56.8)	
ypN1	2 (13.3)	12 (32.4)	
ypN2	0	4 (10.8)	
Distal resection margin (cm)	1.5 (0.2–6.0)	2.0 (0.1–5.0)	0.121^†^
Circumferential resection margin involvement			0.498^¶^
Yes	1 (6.7)	1 (2.7)	
No	14 (93.3)	36 (97.3)	
Harvested lymph nodes	13 (1–18)	13 (1–35)	0.255^†^
Lymphovascular invasion			0.04^¶,**^
Yes	2 (13.3)	16 (43.2)	
No	13 (86.7)	21 (56.8)	
Perineural invasion			0.3^¶^
Yes	2 (13.3)	12 (32.4)	
No	13 (86.7)	25 (67.6)	

Data are presented as median (range) for continuous variables and *n* (%) for categorical variables

^†^Mann–Whitney test; ^¶^chi-square test or Fisher’s exact test; ^**^statistical significance (*P* < 0.05)

*MG*, mistletoe group; *NMG*, non-mistletoe group; *NCRT*, neoadjuvant chemoradiotherapy

Tumor responses were significantly better in the MG than in the NMG in terms of the pCR rate (53.3% vs. 21.6%, *P* = 0.044), TRG (good response 66.7% vs. 32.4%, *P* = 0.024), T downstaging (86.7% vs. 43.2%, *P* = 0.004), and overall TNM downstaging (86.7% vs. 56.8%, *P* = 0.040). N downstaging showed no statistically significant difference between the two groups (93.3% vs. 78.4%, *P* = 0.257). Table 5 provides the details of the tumor responses.

**Table 5:** Tumor responses between the mistletoe and non-mistletoe groups

Variables	MG (*n* = 15)	NMG (*n* = 37)	*P*-value
pCR			0.044^¶,**^
Yes	8 (53.3)	8 (21.6)	
No	7 (46.7)	29 (78.4)	
TRG (AJCC)			0.024^¶,**^
Good responder (0–1)	10 (66.7)	12 (32.4)	
Poor responder (2–3)	5 (33.3)	25 (67.6)	
T downstaging			0.004^¶,**^
Yes	13 (86.7)	16 (43.2)	
No	2 (13.3)	21 (56.8)	
N downstaging			0.257^¶^
Yes	14 (93.3)	29 (78.4)	
No	1 (6.7)	8 (21.6)	
Overall TNM downstaging			0.040^¶,**^
Yes	13 (86.7)	21 (56.8)	
No	2 (13.3)	16 (43.2)	

Data are presented as *n* (%)

^¶^Chi-square test or Fisher’s exact test; ^**^statistically significant (*P* < 0.05)

*MG*, mistletoe group; *NMG*, non-mistletoe group; *pCR*, pathologic complete response; *TRG*, tumor regression grade; *AJCC*, American Joint Committee on Cancer; *TNM*, tumor–node–metastasis

### TUNEL assay and western blot analysis

**Fig. 1:**
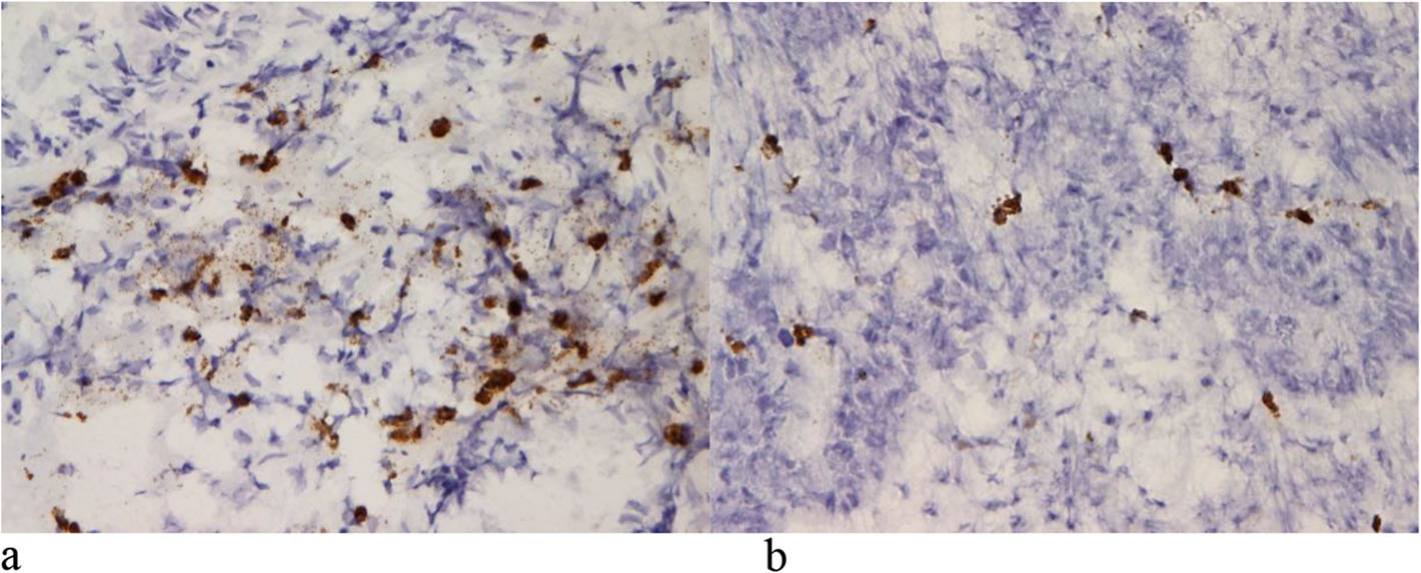
Terminal deoxynucleotidyl transferase-mediated dUTP-biotin nick-end-labeling (TUNEL) staining using 3,3′-diaminobenzidine tetrachloride to assess apoptosis. **a** More TUNEL-positive tumor cells were observed in samples from the mistletoe group (MG) than in the non-mistletoe group (NMG). **b** Representative TUNEL-stained samples from the non-mistletoe group (NMG)

**Fig. 2:**
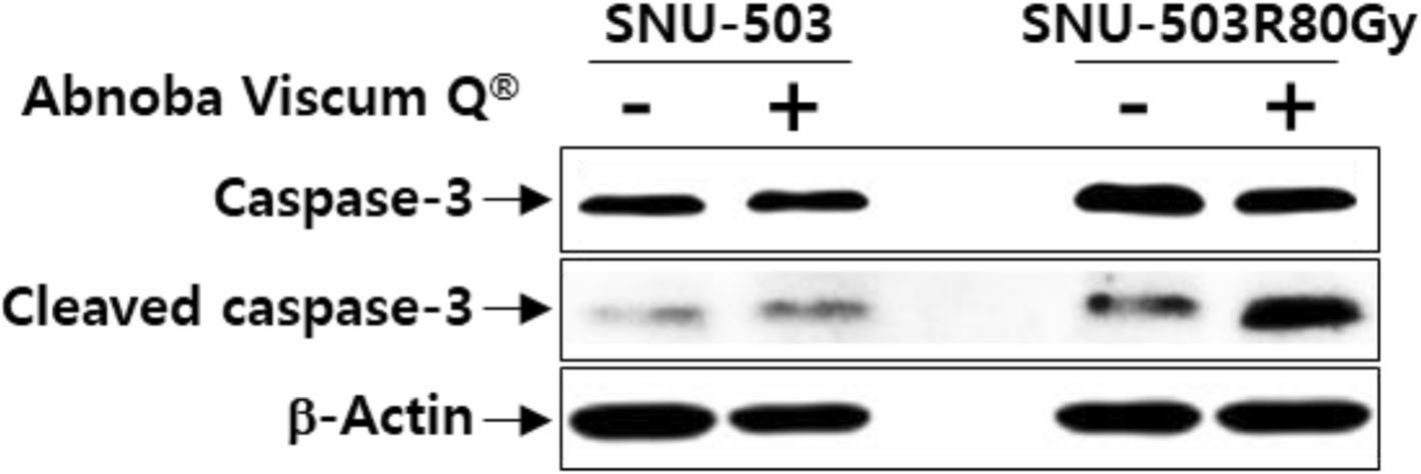
Western blot analysis. The level of cleaved caspase-3, a marker of apoptosis, was increased in two rectal cancer cell lines SNU-503 and SNU-503R80Gy following treatment with Abnoba Viscum Q® extract

There were more TUNEL-positive tumor cells in samples from the MG than in those from the NMG (Fig. 1). The level of cleaved caspase-3 (active form) was increased in the two rectal cancer cell lines—SNU-503R80Gy (radioresistant rectal cancer) and SNU-503—following treatment with Abnoba Viscum Q® than that before the treatment (Fig. 2).

## Discussion

To the best of our knowledge, this is the first study to analyze the response of locally advanced rectal tumors to NCRT and mistletoe extract. The results of the study are promising since it demonstrates that administration of mistletoe extract in combination with NCRT showed better outcomes for tumor responses, including T stage, lymphovascular invasion, TNM stage, TRG, and pCR. These results suggest that mistletoe extract may lead to an overall improvement in the oncologic outcome in patients with locally advanced rectal cancer.

Chemoradiotherapy for rectal cancer usually requires locoregional therapy because of the relatively high risk of locoregional recurrence compared with colon cancer. Preoperative irradiation is more effective than postoperative since tumor oxygenation is better preoperatively [20]. NCRT decreases locoregional recurrence and is associated with reduced toxicity compared to postoperative chemoradiotherapy [2, 21]. Therefore, NCRT with 5-FU or capecitabine is usually the standard therapy for locally advanced rectal cancer before radical surgery [22, 23]. NCRT before surgery for stage II or III rectal cancer diminishes locoregional recurrence and reduces the toxicity of chemoradiation [2, 24]. However, despite these treatment approaches, the 5-year incidence of locoregional recurrence of rectal cancer ranges from 6.0 to 10.7% [2, 8, 25, 26]. Tumor downstaging and pCR after NCRT facilitate increased overall survival and disease-free survival, including locoregional therapy [4–8]. Therefore, it is crucial to increase tumor downstaging and pCR to achieve good oncological results in patients with rectal cancer treated with NCRT.

Curative surgery is recommended within 5–12 weeks after the completion of NCRT. Longer intervals (more than the classical 6–8 weeks) from the completion of NCRT increase the rate of pCR by 6% in rectal cancer [27]. An interval of over 8 weeks is associated with increased odds of pCR compared with an interval of 6–8 weeks [28]. The median intervals to surgery in the current study were 8.6 and 8.7 weeks in the MG and the NMG, respectively.

Mistletoe is a parasitic plant found in trees. Mistletoe extract was first introduced for cancer treatment by Rudolf Steiner [29]. Cancerostatic protein components from *Viscum album* were later identified as cytotoxic viscotoxins and mistletoe lectins [30]. Mistletoe extract induces apoptosis and cytotoxicity in cancer cells [31]. It also stimulates immunocompetent cells to promote immunomodulation as mistletoe lectin is a beta-galactoside-specific lectin [32]. The most important pharmacological components of mistletoe extract are viscotoxins and lectins, which exhibit antitumor and immunomodulatory effects. Previous studies have shown several synergistic effects of chemotherapy with mistletoe extract and antitumor drugs. Mistletoe lectin in combination with rosiglitazone demonstrated enhanced antiproliferative efficacy for malignant melanoma [33]. Sabová et al. studied the cytotoxic and apoptotic effects of mistletoe extract in combination with doxorubicin on Jurkat cells and observed a dose-dependent DNA fragmentation [34]. Schötterl et al., using in vitro and in vivo assays, investigated the synergistic effect of mistletoe-based drugs with TMZ-based chemoradiation in the treatment of glioblastoma [35]. The combined treatment showed additive effects in reducing the survival of the tested tumor cell lines in clonogenic assays. Based on the results of the available literature, we believed that mistletoe extract may act synergistically with NCRT to promote tumor regression. In the current study, the TUNEL assay was used to confirm increased cell death and apoptosis after curative surgery in patients receiving mistletoe extract. Cleavage of caspase-3 is one of the hallmarks of apoptosis. Western blot analysis showed that the level of cleaved caspase protein was increased in the two rectal cancer cell lines tested following treatment with Abnoba Viscum Q® extract, indicating that apoptosis was further increased by the extract.

Abnoba Viscum Q®, which showed a radioprotective effect similar to that of amifostine, has been approved as a clinical radioprotectant [36]. The viscotoxin in the mistletoe extract has potential antioxidant activity [37]. Mistletoe extract when combined with chemotherapy and/or radiotherapy for stage I–III colorectal cancer led to a reduction in adverse reactions after adjuvant therapy and better oncologic outcomes in terms of disease-free survival [38]. In the present study, Abnoba Viscum Q® was used which we anticipated to have dual effects, namely, radio protectivity and tumor regression, in patients with locally advanced rectal cancer.

Tumor regression after conventional NCRT occurs in most patients, and approximately 12–38% of patients can achieve pCR [39]. To enhance the pathologic tumor response and improve locoregional therapy, more toxic chemotherapeutic agents and biologics, including oxaliplatin or targeted agents, have been added to the conventional NCRT regime to increase pCR and thereby improve oncologic outcomes, including reduced recurrence in patients with rectal cancer. However, oxaliplatin as a radiation sensitizer does not improve clinical outcomes, including pCR and patient survival, but increases chemotoxicity [10, 11, 40–42]. Clinical studies with targeted agents, such as cetuximab, panitumumab, and bevacizumab, in NCRT for advanced rectal cancer reported that this approach does not increase pCR, but leads to a significant increase in toxicities [12, 13, 43, 44]. Therefore, a new, less toxic agent needs to be identified that could enhance tumor regression in patients with rectal cancer. Recently, metformin with NCRT was examined in rectal cancer, and a good tumor response and cancer-specific survival rate, as well as a lower risk of cancer recurrence, was reported [45]. Herein, we used mistletoe extract, which has been approved by the Korean Food and Drug Administration, as an anti-malignant drug for NCRT in advanced rectal cancer to enhance the tumor response. The pCR rates in the MG and the NMG were 53.3% and 21.6%, respectively, indicating that mistletoe extract almost doubled the effect of NCRT, with statistical significance. The T downstaging rates in the MG and the NMG were 86.7% and 43.2%, respectively, and the overall downstaging rates were 86.7% and 56.8%, respectively, with statistically significant differences. Further, lymphovascular invasion was less frequently detected in the MG than in the NMG (13.3% vs. 32.4%, *P* = 0.04). Although this study is retrospective and not randomized, the results are very encouraging as compared to those of previous studies [10, 11, 28, 39–41]. We expect to further improve the oncological outcomes in the future by using additional mistletoe extract for NCRT in rectal cancer based on these pathological findings.

Patients receiving NCRT with toxic chemotherapeutic agents, including oxaliplatin or bevacizumab, experience increased rates of grade 3 or 4 toxicity [10–12, 42, 44]. However, mistletoe extract combined with chemotherapeutic regimens can improve the health-related quality of life in patients with solid tumors [16, 46]. This extract reduces the incidence of gastrointestinal adverse effects, such as diarrhea, in patients with gastric cancer during adjuvant chemotherapy [16]. In a previous randomized comparative study by Pelzer et al. in patients with breast cancer, the mistletoe group showed a positive trend towards less neutropenia (*P* = 0.178), had significantly reduced tumor-induced pain (*P* < 0.0001), and loss of appetite (*P* = 0.047) [47]. In the present study, all adverse events in the MG, including gastrointestinal symptoms, oral mucositis, and peripheral neuropathy, were of grade 1. Only patients in the NMG reported grade 2 neutropenia and grade 3 anemia; however, pruritus was observed in the MG. There were no significant differences between the MG and the NMG in the occurrence of adverse events other than pruritis. Collectively, the results show that mistletoe extract is a safer and less toxic agent for the treatment of patients with rectal cancer receiving NCRT. Mistletoe extracts may thus help reduce chemotherapy-induced toxicity and enhance tolerability to cancer treatment.

In patients receiving NCRT for rectal cancer, the better the tumor regression, the better the patient’s prognosis is. Long-term oncological results of clinical complete response (cCR) after NCRT in rectal cancer are similar to pCR [48]. Watch-and-wait strategy mediating a systematic and strict follow-up in cCR patients following NCRT could accomplish similar clinical outcomes as those with surgery [49]. As Abnoba is a representative anticancer complementary agent with fewer side effects, its combination with NCRT could be expected to enhance tumor regression and confer good long-term clinical results. Furthermore, if a higher cCR rate is obtained with NCRT for rectal cancer, an effective approach can be designed for a non-operative management using the watch-and-wait strategy.

A few limitations of this study require particular mention. First, the number of patients who received NCRT needs to be large so as to statistically analyze the effect of mistletoe extract on locally advanced rectal cancer. We experienced practical limitations in terms of financial constraints on patients since mistletoe extract is not reimbursed under the national insurance system in Korea. Second, this was a retrospective, non-randomized study. Third, the downstaging assessment of rectal tumors after NCRT has unavoidable limitations in terms of accuracy because the evaluation of preoperative tumor staging was based on imaging studies. Fourth, the mechanism of mistletoe extract underlying tumor regression has not been investigated in detail. Nonetheless, this study is significant in that it is the first to demonstrate better outcomes following the use of mistletoe extract in combination with NCRT for locally advanced rectal cancer.

## Conclusions

NCRT in combination with mistletoe (Abnoba Viscum Q®) extract led to a better tumor response for rectal cancer than conventional chemoradiation alone. Nevertheless, further large prospective randomized studies with long-term follow-up are required to confirm the effectiveness of the extract in chemoradiation for patients with locally advanced rectal cancer.

## Data Availability

All data are available from the corresponding author on reasonable request.

## Abbreviations

NCRT: Neoadjuvant chemoradiotherapy
pCR: Pathologic complete response
cCR: Clinical complete response
AJCC: American Joint Committee on Cancer
yp: Pathological status after NCRT
CA 19-9: Carbohydrate antigen 19-9
MG: Mistletoe group
NMG: Non-mistletoe group
TUNEL: Terminal deoxynucleotidyl transferase-mediated dUTP-biotin nick-end labeling
TRG: Tumor regression grade
5-FU: 5-Fluorouracil
TdT: Terminal deoxynucleotidyl transferase
TNM: Tumor–node–metastasis
MRF: Mesorectal fascia
CT: Computed tomography
MRI: Magnetic resonance imaging
AV: Anal verge

## References

[1] Bray F, Ferlay J, Soerjomataram I, Siegel RL, Torre LA, Jemal A (2018). Global cancer statistics 2018: GLOBOCAN estimates of incidence and mortality worldwide for 36 cancers in 185 countries. CA Cancer J Clin..

[2] Sauer R, Becker H, Hohenberger W, Rodel C, Wittekind C, Fietkau R (2004). Preoperative versus postoperative chemoradiotherapy for rectal cancer. N Engl J Med..

[3] Sauer R, Liersch T, Merkel S, Fietkau R, Hohenberger W, Hess C, Becker H, Raab HR, Villanueva MT, Witzigmann H, Wittekind C, Beissbarth T, Rödel C (2012). Preoperative versus postoperative chemoradiotherapy for locally advanced rectal cancer: results of the German CAO/ARO/AIO-94 randomized phase III trial after a median follow-up of 11 years. J Clin Oncol..

[4] Xu L, Cai S, Xiao T, Chen Y, Qiu H, Wu B, Lin G, Sun X, Lu J, Zhou W, Xiao Y (2017). Prognostic significance of tumour regression grade after neoadjuvant chemoradiotherapy for a cohort of patients with locally advanced rectal cancer: an 8-year retrospective single-institutional study. Colorectal Dis..

[5] Jalil O, Claydon L, Arulampalam T (2015). Review of neoadjuvant chemotherapy alone in locally advanced rectal cancer. J Gastrointest Cancer..

[6] Theodoropoulos G, Wise WE, Padmanabhan A, Kerner BA, Taylor CW, Aguilar PS, Khanduja KS (2002). T-level downstaging and complete pathologic response after preoperative chemoradiation for advanced rectal cancer result in decreased recurrence and improved disease-free survival. Dis Colon Rectum..

[7] Maas M, Nelemans PJ, Valentini V, Das P, Rödel C, Kuo L-J, Calvo FA, García-Aguilar J, Glynne-Jones R, Haustermans K, Mohiuddin M, Pucciarelli S, Small W, Suárez J, Theodoropoulos G, Biondo S, Beets-Tan RGH, Beets GL (2010). Long-term outcome in patients with a pathological complete response after chemoradiation for rectal cancer: a pooled analysis of individual patient data. Lancet Oncol..

[8] Roh MS, Colangelo LH, O'Connell MJ, Yothers G, Deutsch M, Allegra CJ, Kahlenberg MS, Baez-Diaz L, Ursiny CS, Petrelli NJ, Wolmark N (2009). Preoperative multimodality therapy improves disease-free survival in patients with carcinoma of the rectum: NSABP R-03. J Clin Oncol..

[9] Lin H, Wang L, Zhong X, Zhang X, Shao L, Wu J (2021). Meta-analysis of neoadjuvant chemotherapy versus neoadjuvant chemoradiotherapy for locally advanced rectal cancer. World J Surg Oncol..

[10] Allegra CJ, Yothers G, O'Connell MJ, Beart RW, Wozniak TF, Pitot HC (2015). Neoadjuvant 5-FU or capecitabine plus radiation with or without oxaliplatin in rectal cancer patients: a phase III randomized clinical trial. J Natl Cancer Inst.

[11] O'Connell MJ, Colangelo LH, Beart RW, Petrelli NJ, Allegra CJ, Sharif S, Pitot HC, Shields AF, Landry JC, Ryan DP, Parda DS, Mohiuddin M, Arora A, Evans LS, Bahary N, Soori GS, Eakle J, Robertson JM, Moore DF, Mullane MR, Marchello BT, Ward PJ, Wozniak TF, Roh MS, Yothers G, Wolmark N (2014). Capecitabine and oxaliplatin in the preoperative multimodality treatment of rectal cancer: surgical end points from National Surgical Adjuvant Breast and Bowel Project trial R-04. J Clin Oncol..

[12] Landry JC, Feng Y, Prabhu RS, Cohen SJ, Staley CA, Whittington R, Sigurdson ER, Nimeiri H, Verma U, Benson AB (2015). Phase II trial of preoperative radiation with concurrent capecitabine, oxaliplatin, and bevacizumab followed by surgery and postoperative 5-fluorouracil, leucovorin, oxaliplatin (FOLFOX), and bevacizumab in patients with locally advanced rectal cancer: 5-year clinical outcomes ECOG-ACRIN Cancer Research Group E3204. Oncologist..

[13] Pinto C, Di Bisceglie M, Di Fabio F, Bochicchio A, Latiano T, Cordio S (2018). Phase II study of preoperative treatment with external radiotherapy plus panitumumab in low-risk, locally advanced rectal cancer (RaP Study/STAR-03). Oncologist..

[14] Stan RL, Hangan AC, Dican L, Sevastre B, Hanganu D, Catoi C, Sarpataki O, Ionescu C (2013). Comparative study concerning mistletoe viscotoxins antitumor activity. Acta Biol Hung..

[15] Yoon TJ, Yoo YC, Kang TB, Song SK, Lee KB, Her E, Song KS, Kim JB (2003). Antitumor activity of the Korean mistletoe lectin is attributed to activation of macrophages and NK cells. Arch Pharm Res..

[16] Kim KC, Yook JH, Eisenbraun J, Kim BS, Huber R (2012). Quality of life, immunomodulation and safety of adjuvant mistletoe treatment in patients with gastric carcinoma - a randomized, controlled pilot study. BMC Complement Altern Med..

[17] Thronicke A, Oei SL, Merkle A, Matthes H, Schad F (2018). Clinical safety of combined targeted and *Viscum album* L. therapy in oncological patients. Medicines (Basel).

[18] Kienle GS, Kiene H (2010). Review article: Influence of *Viscum album* L (European mistletoe) extracts on quality of life in cancer patients: a systematic review of controlled clinical studies. Integr Cancer Ther..

[19] Trakarnsanga A, Gonen M, Shia J, Nash GM, Temple LK, Guillem JG (2014). Comparison of tumor regression grade systems for locally advanced rectal cancer after multimodality treatment. J Natl Cancer Inst.

[20] Colorectal Cancer Collaborative Group (2001). Adjuvant radiotherapy for rectal cancer: a systematic overview of 8,507 patients from 22 randomised trials. Lancet..

[21] Minsky BD, Cohen AM, Kemeny N, Enker WE, Kelsen DP, Reichman B, Saltz L, Sigurdson ER, Frankel J (1992). Combined modality therapy of rectal cancer: decreased acute toxicity with the preoperative approach. J Clin Oncol..

[22] Hofheinz RD, Wenz F, Post S, Matzdorff A, Laechelt S, Hartmann JT, Müller L, Link H, Moehler M, Kettner E, Fritz E, Hieber U, Lindemann HW, Grunewald M, Kremers S, Constantin C, Hipp M, Hartung G, Gencer D, Kienle P, Burkholder I, Hochhaus A (2012). Chemoradiotherapy with capecitabine versus fluorouracil for locally advanced rectal cancer: a randomised, multicentre, non-inferiority, phase 3 trial. Lancet Oncol..

[23] O’Connell MJ, Martenson JA, Wieand HS, Krook JE, Macdonald JS, Haller DG (1994). Improving adjuvant therapy for rectal cancer by combining protracted-infusion fluorouracil with radiation therapy after curative surgery. N Engl J Med..

[24] Rahbari NN, Elbers H, Askoxylakis V, Motschall E, Bork U, Buchler MW (2013). Neoadjuvant radiotherapy for rectal cancer: meta-analysis of randomized controlled trials. Ann Surg Oncol..

[25] Gérard JP, Conroy T, Bonnetain F, Bouche O, Chapet O, Closon-Dejardin MT (2006). Preoperative radiotherapy with or without concurrent fluorouracil and leucovorin in T3-4 rectal cancers: results of FFCD 9203. J Clin Oncol..

[26] Bosset JF, Collette L, Calais G, Mineur L, Maingon P, Radosevic-Jelic L, Daban A, Bardet E, Beny A, Ollier JC (2006). Chemotherapy with preoperative radiotherapy in rectal cancer. N Engl J Med..

[27] Petrelli F, Sgroi G, Sarti E, Barni S (2016). Increasing the interval between neoadjuvant chemoradiotherapy and surgery in rectal cancer: a meta-analysis of published studies. Ann Surg..

[28] Probst CP, Becerra AZ, Aquina CT, Tejani MA, Wexner SD, Garcia-Aguilar J, Remzi FH, Dietz DW, Monson JR, Fleming FJ, Consortium for Optimizing the Surgical Treatment of Rectal Cancer (OSTRiCh) (2015). Extended intervals after neoadjuvant therapy in locally advanced rectal cancer: the key to improved tumor response and potential organ preservation. J Am Coll Surg..

[29] Steiner R (1985). Geisteswissenschaft und Medizin.

[30] Vester F, Nienhaus J (1965). Cancerostatic protein components from *Viscum album*. Experientia..

[31] Büssing A (1996). Induction of apoptosis by the mistletoe lectins: a review on the mechanisms of cytotoxicity mediated by *Viscum album* L. Apoptosis..

[32] Hajto T, Hostanska K, Frei K, Rordorf C, Gabius HJ (1990). Increased secretion of tumor necrosis factors alpha, interleukin 1, and interleukin 6 by human mononuclear cells exposed to beta-galactoside-specific lectin from clinically applied mistletoe extract. Cancer Res..

[33] Freudlsperger C, Dahl A, Hoffmann J, Reinert S, Schumacher U (2010). Mistletoe lectin-I augments antiproliferative effects of the PPAR gamma agonist rosiglitazone on human malignant melanoma cells. Phytother Res..

[34] Sabova L, Pilatova M, Szilagyi K, Sabo R, Mojzis J (2010). Cytotoxic effect of mistletoe (*Viscum album* L.) extract on Jurkat cells and its interaction with doxorubicin. Phytother Res..

[35] Schotterl S, Miemietz JT, Ilina EI, Wirski NM, Ehrlich I, Gall A (2019). Mistletoe-based drugs work in synergy with radio-chemotherapy in the treatment of glioma *in vitro* and *in vivo* in glioblastoma bearing mice. Evid Based Complement Alternat Med..

[36] Rim CH, Koun S, Park HC, Lee S, Kim CY (2019). Radioprotective effects of mistletoe extract in zebrafish embryos in vivo. Int J Radiat Biol..

[37] Orhan DD, Aslan M, Sendogdu N, Ergun F, Yesilada E (2005). Evaluation of the hypoglycemic effect and antioxidant activity of three *Viscum album* subspecies (European mistletoe) in streptozotocin-diabetic rats. J Ethnopharmacol..

[38] Friedel WE, Matthes H, Bock PR, Zanker KS (2009). Systematic evaluation of the clinical effects of supportive mistletoe treatment within chemo- and/or radiotherapy protocols and long-term mistletoe application in nonmetastatic colorectal carcinoma: multicenter, controlled, observational cohort study. J Soc Integr Oncol..

[39] Cui J, Fang H, Zhang L, Wu YL, Zhang HZ (2016). Advances for achieving a pathological complete response for rectal cancer after neoadjuvant therapy. Chronic Dis Transl Med..

[40] Aklilu M, Eng C (2011). The current landscape of locally advanced rectal cancer. Nat Rev Clin Oncol..

[41] Gérard JP, Azria D, Gourgou-Bourgade S, Martel-Lafay I, Hennequin C, Etienne PL, Vendrely V, François E, de la Roche G, Bouché O, Mirabel X, Denis B, Mineur L, Berdah JF, Mahé MA, Bécouarn Y, Dupuis O, Lledo G, Seitz JF, Bedenne L, Juzyna B, Conroy T (2012). Clinical outcome of the ACCORD 12/0405 PRODIGE 2 randomized trial in rectal cancer. J Clin Oncol..

[42] Lee WS, Baek JH, Shin DB, Sym SJ, Kwon KA, Lee KC, Lee SH, Jung DH (2013). Neoadjuvant treatment of mid-to-lower rectal cancer with oxaliplatin plus 5-fluorouracil and leucovorin in combination with radiotherapy: a Korean single center phase II study. Int J Clin Oncol..

[43] Dewdney A, Cunningham D, Tabernero J, Capdevila J, Glimelius B, Cervantes A, Tait D, Brown G, Wotherspoon A, Gonzalez de Castro D, Chua YJ, Wong R, Barbachano Y, Oates J, Chau I (2012). Multicenter randomized phase II clinical trial comparing neoadjuvant oxaliplatin, capecitabine, and preoperative radiotherapy with or without cetuximab followed by total mesorectal excision in patients with high-risk rectal cancer (EXPERT-C). J Clin Oncol..

[44] Helbling D, Bodoky G, Gautschi O, Sun H, Bosman F, Gloor B, Burkhard R, Winterhalder R, Madlung A, Rauch D, Saletti P, Widmer L, Borner M, Baertschi D, Yan P, Benhattar J, Leibundgut EO, Bougel S, Koeberle D (2013). Neoadjuvant chemoradiotherapy with or without panitumumab in patients with wild-type KRAS, locally advanced rectal cancer (LARC): a randomized, multicenter, phase II trial SAKK 41/07. Ann Oncol..

[45] Kim JM, Park JW, Lee JH, Park YH, Park SJ, Cheon JH, Kim WH, Kim TI (2020). Survival benefit for metformin through better tumor response by neoadjuvant concurrent chemoradiotherapy in rectal cancer. Dis Colon Rectum..

[46] Lange-Lindberg AM, Velasco Garrido M, Busse R (2006). Mistletoe treatments for minimising side effects of anticancer chemotherapy. GMS Health Technol Assess.

[47] Pelzer F, Troger W, Nat DR (2018). Complementary treatment with mistletoe extracts during chemotherapy: safety, neutropenia, fever, and quality of life assessed in a randomized study. J Altern Complement Med..

[48] Coraglio MF, Eleta MA, Kujaruk MR, Oviedo JH, Roca EL, Masciangioli GA, Mendez G, Iseas IS (2020). Analysis of long-term oncological results of clinical versus pathological responses after neoadjuvant treatment in locally advanced rectal cancer. World J Surg Oncol..

[49] Zhao GH, Deng L, Ye DM, Wang WH, Yan Y, Yu T (2020). Efficacy and safety of wait and see strategy versus radical surgery and local excision for rectal cancer with cCR response after neoadjuvant chemoradiotherapy: a meta-analysis. World J Surg Oncol..

